# Exploring Desired Features of Mobile Health Apps for Patients With Diabetes to Enhance Engagement and Self-Management: Qualitative Study

**DOI:** 10.2196/69176

**Published:** 2025-08-14

**Authors:** Arkers Kwan Ching Wong, Ying Nan, Bing Xiang Yang, Yanqun Liu

**Affiliations:** 1 Hong Kong Polytechnic University, School of Nursing, 1 Cheong Wan Road, Kowloon, Hong Kong, China (Hong Kong), 852 34003805; 2 Southern Medical University, School of Nursing, Guangzhou, China; 3 Wuhan University, School of Nursing, Wuhan, China

**Keywords:** diabetes mellitus, mHealth, mobile app, self-management, smartphone

## Abstract

**Background:**

Diabetes mellitus (DM) is a chronic condition requiring effective self-management to maintain glycemic control and prevent complications. Mobile health (mHealth) apps offer potential solutions by providing real-time monitoring, personalized feedback, and educational resources. However, their long-term adoption is hindered by a lack of user involvement in the development process and insufficient cultural adaptation. This study aims to explore the perspectives of patients with DM in Hong Kong on the functionalities and features of mHealth apps, highlighting the importance of tailoring these apps to meet local cultural needs.

**Objective:**

The objective of this study is to understand the views of patients with DM on the development of mHealth apps and the demand for app functions in order to provide a basis for the development of DM prevention apps.

**Methods:**

This descriptive qualitative study conducted semi-structured interviews with 10 patients with DM attending a District Health Centre in Hong Kong in May 2024, using a purposive sampling strategy. The transcribed data were analyzed by the inductive content analytical method, and themes were extracted with the aid of NVivo (version 15.0; QSR International) software.

**Results:**

In total, 7 key themes were identified: accurate information resources, automatic tracking and monitoring of health metrics, reminders, personalized customization options, intuitive usability, efficient data-sharing capabilities, and interactive design. Additionally, the study emphasizes the importance of cultural adaptation and the potential of artificial intelligence–enabled mHealth apps to enhance personalized information delivery. Ensuring the credibility and professionalism of information sources is also essential.

**Conclusions:**

The results provide valuable insights for enhancing the self-management capabilities of patients with DM and inform the future development of mHealth apps focused on DM prevention.

## Introduction

Diabetes mellitus (DM) is a chronic disease characterized by high incidence, disability rates, and mortality, posing significant threats to human health and representing a major public health concern [1]. Effective self-management practices—such as tracking blood glucose levels, adhering to medication or insulin therapies, monitoring nutrition, and engaging in regular physical activity—are crucial for maintaining glycemic control and preventing diabetes-related complications [2]. The advent of mobile health (mHealth) smartphone apps has introduced an innovative approach for patients with DM to enhance their self-care capabilities [3-5]. However, despite their proven effectiveness in DM management, the long-term use of these mHealth apps has been hindered by high attrition rates [6]. This attrition has been attributed to insufficient consideration of end-users’ preferences and self-management needs, which are vital for fostering long-term engagement and informing the development of effective mHealth solutions [7,8].

To date, limited studies have explored patients’ usage patterns, feature preferences, and recommendations for essential DM mHealth apps. Existing evidence suggests diverse views among patients regarding the key functionalities required to support their self-care needs. While some studies highlight preferences for data recording, social coaching, reminders, and remote collaboration with health care professionals [9,10], others emphasize the importance of carbohydrate counting, glucose tracking, and activity monitoring [11,12]. Additionally, some studies on patient needs did not include participants who had actual app usage, limiting the applicability of their findings [13].

Prior research has emphasized the importance of cultural adaptation in the success of mHealth apps [14]. Insufficient tailoring of app functionalities to align with cultural norms, values, and preferences has posed significant challenges for DM self-management, reducing patients’ overall acceptability, adherence, and the effectiveness of such interventions [15]. Therefore, it is crucial to adjust self-management mHealth apps for patients with DM based on distinct cultural, lifestyle, and dietary habits across different regions and ethnicities. Most existing research has been conducted in Western countries, such as the United Kingdom [11,16,17] and the United States [13,14], as well as in Mainland China [10,18]. Very few studies have focused on the health care system and cultural landscape of Hong Kong.

Hong Kong, as a densely populated and highly urbanized city with a blend of Eastern and Western influences, exhibits distinctive dietary habits, lifestyle patterns, and linguistic frameworks. For example, the popularity of Cantonese cuisine alongside Western fast food has increased the demand for dietary caloric conversion features within mHealth apps. Furthermore, the necessity for multilingual support—including Cantonese, English, and Mandarin—highlights the importance of incorporating multilingual design and voice recognition capabilities to enhance user accessibility and engagement.

In light of these contextual factors, this qualitative study aims to explore the requirements and preferences of patients with DM in Hong Kong for the functions and design of mHealth apps. The insights gained are expected to enhance their self-management capabilities and provide valuable references to inform the design and development of such apps.

## Methods

### Study Design

This study used a descriptive qualitative research design, grounded in the philosophical tenets of naturalistic inquiry [19]. This qualitative approach uses everyday language and low-inference interpretative processes to represent reality by describing experiences or events [20]. It is suitable for informing diverse practical applications, such as clinical interventions, scoring systems, needs assessments, questionnaire development, and surveys [19]. Therefore, this method was well-suited to our aim of gaining rich, contextualized insights into the requirements and preferences of patients with DM for mHealth app features.

We collected data using semi-structured, in-depth interviews. The interview guide was developed based on prior experience and a review of relevant literature, aligned with the study’s objectives. Before the formal interviews, 2 patients with DM were pre-interviewed, and the interview outline was adjusted according to the pre-interview analysis results. The formal interview outline is presented in Textbox 1. The study outcomes were reported according to the Consolidated Criteria for Reporting Qualitative Research [21].

Textbox 1. **Semi-structured interview guide.**

1. Could you share some of your experiences with your diabetes mellitus (DM) condition and the challenges you’ve faced in self-management?

2. Do you currently use an mHealth app to manage your DM, or have you used one in the past?

 a. Which mHealth management apps have you used? How long and how often have you used them?

 b. What features do you find particularly useful in these apps? Are there any features that are not very useful and could be improved further?

3. We are aiming to develop an mHealth app to help manage your daily health. What features would you like to see in it?

4. Is there anything else you would like to add on the topics we have discussed today?

### Participants and Recruitment

A purposive sampling approach was used to select participants diagnosed with DM for the qualitative interviews. Eligible patients were recruited from the Hong Kong District Health Centre in May 2024. Recruitment posters were displayed at the center to solicit participant involvement. A research assistant established initial phone contact with eligible patients, introducing the study, explaining the consent procedures, and informing them about the duration of the in-depth interviews. Patients willing to participate were scheduled for in-person interviews conducted on-site at the center. All eligible participants who initially consented completed the scheduled interview sessions without dropouts or refusals.

The inclusion criteria were as follows: (1) diagnosed with DM according to the 2019 WHO diagnostic criteria; (2) aged 18‐80 years; (3) no cognitive impairment and able to communicate effectively with health care professionals; (4) signed informed consent and agreed to participate in the study. The exclusion criteria were as follows: (1) patients with severe complications or significant dysfunction of other major organs; (2) patients with incomplete data or who withdrew from the study.

The sample size was determined based on the principle of information saturation. After the 8th interview, no new themes emerged, and 2 additional interviews were conducted to confirm saturation, ultimately resulting in a total of 10 participants. To protect patient privacy, these individuals have been labeled as P1 through P10. Demographic information was collected for each participant before initiating an interview session and is reported in Table 1.

**Table 1.** Demographic and health characteristics of total participants.

Characteristic	Participants, n (%)	
Age (years)		
60‐75	10 (100)	
76 or older	0 (0)	
Gender		
Male	9 (90)	
Female	1 (10)	
Education		
Primary school	1 (10)	
High school	6 (60)	
Tertiary education	3 (30)	
Employment status		
Employed	2 (20)	
Unemployed	0 (0)	
Retired	8 (80)	

### Data Collection

Face-to-face interviews, each lasting 60 minutes, were conducted in an independent activity room at the Center, free from external distractions. The research team, led by a principal investigator and supported by 2 research assistants, conducted all interview sessions and collected field notes throughout the process. The team had undergone comprehensive training in qualitative research methods, equipping them with the necessary skills and expertise for this study.

Each interview commenced with a brief introduction. Interviews were recorded with participant consent and guided by a pre-tested, semi-structured protocol. No other individuals were present during the interviews. Participants were not involved in the design, conduct, or reporting of the dissemination of this research. All interviews were conducted in a single session, with no repeat interviews.

Prior to the interviews, participants were informed of the study’s purpose and assured of privacy protections. The main topics included factors influencing effective DM management and perceived useful features of mHealth apps for improving self-care. During the interviews, researchers actively listened, used appropriate questioning techniques, and promptly recorded key information. They also observed and documented participants’ nonverbal cues, such as pauses, smiles, body language, and mood changes. After each session, a reflective diary was written to identify and correct any issues for the next interview.

### Data Analysis

All interviews were audio-recorded and transcribed verbatim. Inductive content analysis was applied to examine the data at both the manifest and latent levels, following the methodology outlined by Elo and Kyngäs [22], which involved open coding, category development, and abstraction. This analytical process was completed using NVivo (version 15.0; QSR International) software. Initially, 2 researchers (NY and WAKC) iteratively reviewed the transcribed text. The analysis began with open coding, where the researchers generated descriptive notes directly in the margins, which were then transferred to a coding sheet to generate subcategories. Following the open coding, the lists of resulting subcategories were grouped into main categories based on both differences and similarities. This analytical process involved ongoing, iterative discussions with the research team to refine the codes and categories, aiming to enhance the overall trustworthiness of the study’s findings. To further ensure credibility, 2 researchers independently coded the data, followed by peer debriefing meetings with the research team to refine the coding framework. An audit trail documenting analytical decisions was maintained to promote dependability. Transferability was supported by detailed reporting of participant demographics and rich contextual descriptions. Quality assurance was incorporated through methodological rigor, including researcher training, data triangulation, and audit trail maintenance.

Data from 7 principal codes and 12 subcodes were assembled into the interest domain (categorization). Data from the remaining codes were considered not relevant to the research aim and were therefore treated as dross (Table 2). The informants were not invited to comment on the transcriptions or provide feedback on the findings. Although participants discussed the functions they valued in an mHealth app, formal prioritization techniques, such as structured ranking exercises, were not used. Instead, relative priorities were inferred based on the frequency and emphasis with which different functions were mentioned during the interviews.

**Table 2.** Desired features and functionality for diabetes mellitus (DM) self-management mHealth app.

Theme or domain	Subtheme
Information resource	Provide clinically validated and comprehensive information covering key self-care aspects such as disease, comorbidities, exercise, and medication.
	Offer guidance to support informed choices regarding diet, fitness, and lifestyle factors.
Automated tracking and monitoring	Track and store historical data, including diet, physical activity, blood glucose levels, and medication usage.
	Automatically convert image data (eg, food photos) into electronic records.
Reminder	Deliver appropriate reminders and alerts for tasks such as monitoring blood sugar regularly, managing medication, and attending health care appointments.
Personalized customization	Provide personalized health recommendations, delivering just-in-time information.
	Automatically generate customized goals, feedback, and action plans based on user input data in a flexible manner.
Intuitive usability	Use simple visual design, and provide interpretation and translation of medical knowledge and test results.
	Incorporate multilingual support and voice recognition capabilities.
Efficient data sharing	Enable seamless integration with the health care system for effective care coordination.
Interactive design	Incorporate in-app peer support communities and forums.
	Enable real-time web-based communication with healthcare coaches.

### Ethical Considerations

This study was approved by the Institutional Review Board of a university in Hong Kong (HSEARS20230705003). All participants provided written informed consent after receiving detailed information about the study’s purpose, procedures, and their right to withdraw at any time without penalty. The present study was conducted according to the principles indicated in the Declaration of Helsinki. These procedures were designed in accordance with institutional data protection policies and ethical standards for qualitative health research. During the interviews, participants were informed that they could refuse to answer any questions they did not wish to answer. To protect their privacy and confidentiality, audio recordings were stored securely, and any identifying information was removed from transcripts. The interview data were anonymized through the coding process to further ensure participant privacy.

## Results

### Participant Characteristics

Among the 10 interviewees, ages ranged from 60 to 75. The majority were retired males with at least a high school education background, and there was only one female participant in the semi-structured interviews.

### Desired Features and Functionality for mHealth App

#### Theme 1: Information Resource

Some participants expressed a desire for mHealth apps to provide clinically validated and comprehensive information covering key areas such as disease knowledge, prevention of comorbidities, dietary information, guidelines for safe and effective exercise, and guidance on medication usage and side effects.

I’m trying to cope with my DM and accept it, but I absolutely want to prevent any complications, particularly with my feet and vision. But the thing is, I’m not totally sure about the specific disease knowledge I need to actually prevent those issues. At the same time, the Internet is full of knowledge, but you don't know which is right and which is wrong.

[Participant #5]

It can be hard to know the amount of sugar in fruits. It’s difficult to figure out how much we’ve actually consumed. Sometimes, we may think that eating fruit is healthy, but we might have actually eaten a lot without realizing it.

[Participant #8]

I’d like to know details about the food, like how many calories are in a packet of fries, and other related information. Especially some classic local foods, you know? Like those egg waffles and curry fish balls. It would be really helpful to have all that in the app. For instance, I looked up the sugar content of an orange, and it was surprisingly high, so I decided not to eat it and gave it to someone else.

[Participant #5]

If I take 10,000 steps, the app can tell me how many calories I have burned. It’s really helpful to have this tool to assist me in making healthier choices.

[Participant #1]

In addition, some participants expressed a need for decision-making support to help them navigate various choices and self-care options for managing their DM effectively. Particularly in events such as social gatherings and celebrations like weddings, the diversity and complexity of food options can be overwhelming. They felt this type of guidance would empower them to make more informed decisions about their treatment, lifestyle modifications, and overall disease management.

I want to go to McDonald’s and eat something, but I don’t know how much I should eat. It would be great if this app had that information. For example, it tells me that I can eat half a burger.

[Participant #5]

When faced with a variety of foods at certain festivals or events, it can be challenging not knowing which options are safe and which require caution regarding portion sizes. It would be helpful if the app could provide guidance on what I should eat.

[Participant #4]

#### Theme 2: Automated Tracking and Monitoring

Participants expressed the need to track and record their food intake, medication, and blood sugar levels, as they often forget these details, which helps them understand their overall health status.

Tracking our diet would be helpful. It would allow us to take control of our food intake and avoid overeating.

[Participant #8]

I often forget to record my blood sugar levels, which should be measured three to four times a day.

[Participant #2]

I hope it can remember my previous blood sugar levels and dietary information, so I can have a better understanding of how my blood sugar changes. It would be great if the app offers options for different time periods like before and after meals, including breakfast. Additionally, I would like to be able to record my dietary habits.

[Participant #6]

For instance, some basic features, like tracking your daily steps, distance, and calories burned, are available on many smartphones. These functions can be helpful to some extent, and you don’t even need to wear a watch.

[Participant #7]

Additionally, participants emphasized the importance of the app automatically converting image data, such as food photos, into electronic records. This automated data tracking functionality would streamline the process of logging dietary intake and other health metrics, making it easier for them to monitor and manage their condition.

Simply take a photo of your meals and food items here to keep a record. It’s a convenient way to keep a record and make a rough estimate.

[Participant #6]

#### Theme 3: Reminder

Participants appreciated the app’s ability to provide timely reminders and alerts to support their self-care, such as prompts to check blood sugar, manage medications, and attend appointments. However, while a “nudge” feature was generally seen as a helpful memory aid, especially for recalling multiple tasks, too many nudges were considered annoying.

If this feature exists, it would be much more convenient. It provides health warnings, reminding you that high levels could be dangerous. The app can also prompt you at 9pm, ‘you should now walk 1000 steps.’Then you go and walk 1000 steps. This is good because it not only helps build bones, but also has benefits for DM.

[Participant #4]

Having a reminder or alert feature is helpful, but if I ignore it today and the same alert appears again tomorrow, I might not pay attention to it. However, if I continue to see the same alert every day, I will eventually take action and follow the advice.

[Participant #3]

It can be annoying when pop-ups appear frequently, so it’s good to have it there when you need it. Like, when you walk past a McDonald’s, it could give a reminder to everyone to try and avoid eating too much fried and greasy stuff. I do not want to have too many cues. Beep, you’re not allowed to eat at McDonald’s. Beep, you can’t eat desserts there.

[Participant #3]

Just one reminder per week, and if you haven’t checked it within that time, it will give you another prompt.

[Participant #2]

#### Theme 4: Personalized Customization

Participants noted that the app should deliver personalized and customizable health recommendations tailored to their individual needs and circumstances. They wanted this guidance to be provided proactively, with the right information at the right time, to empower their decision-making and improve their DM management.

It would be more useful if the app provides personalized recommendations, as general information may not be as helpful since most people are already familiar with it. Everyone has their own unique characteristics, and their treatment plans may differ. The app could use something to provide tailored reminders based on individual circumstances and specific timings.

[Participant #7]

Moreover, the system should generate customized feedback and goals according to their health management standards. If users’ diets or other habits change, the targets and action plan will be promptly updated so they can make self-adjustments to achieve their daily management goals, including food intake, medication, and exercise.

The recommended information can be categorized based on age. For example, let’s say between the ages of 40 and 50, or 50 and 60, the recommended exercise intensity or calorie consumption may differ. In the app, there is a data point that shows how many pounds or how many units of exercise are required to meet the goal based on your age, height, and weight. This way, it can assist people of different ages and body types. You can input your own data, such as weight and height, and the app will calculate accordingly.

[Participant #7]

If your blood glucose levels have been consistently high for a period of time, the app will remind you to pay attention and check if there are any differences in your diet or other aspects. For instance, if your blood glucose levels have risen, the app will provide an analysis to help you reflect on whether you may have consumed excessive food or high-sugar items. It prompts you to consider any changes in your diet or other factors that could have contributed to the increase. It offers monitoring features to help you understand your situation.

[Participant #1]

#### Theme 5: Intuitive Usability

Participants stated that test results and reports can be difficult to comprehend. They hoped that mHealth apps could use simple visual design elements, like charts and graphs, to make information easy to understand.

In the hospital, typically the test results are presented in a report, which helps the doctor to view and understand our health condition. However, these reports can be too complicated for us to comprehend easily. Is it possible to have a visual representation of the trends in values like blood sugar? It would be helpful if we could understand our health status through simple charts or graphs, rather than having to read complex data.

[Participant #7]

After recording these values in the app, there is a feature that shows the trends over time? Usually, these records are for follow-up visits with the doctor, but I often don’t understand them. Can the app provide a visual representation of the trends for easier interpretation?

[Participant #3]

In addition, some participants expressed a need for the app to provide interpretations, translations, and explanations for the data, as they may not fully understand the meaning behind the letters.

In the app, it can provide interpretations and comparisons of medical test results. For example, it uses codes like HbA1C to represent specific meaning, but we might not understand them. Can it provide translations for these codes? The app can also explain common medical terms and abbreviations, such as total cholesterol and triglycerides, to help us better understand the test report. Additionally, it can provide interpretations of common test results, such as the presence of protein in urine, and tell us if they are normal or abnormal. This feature helps us better understand and compare the test results.

[Participant #7]

Beyond these key functions, participants also hoped the app would accommodate diverse user needs through the incorporation of multilingual capabilities. The ability to interact with the app in one’s preferred language was viewed as essential for ensuring accessibility and usability. Moreover, participants highlighted the value of voice recognition features, which would allow them to easily input data and access information using voice commands.

With my vision problems, I was worried the app might be hard for me to use. But the voice commands make it so much more accessible. Saves me a lot of trouble, that’s for sure.

[Participant #2]

As a native Cantonese speaker, being able to use the app in Cantonese is just fantastic. I don’t have to stress about mispronouncing things or using the wrong vocabulary - it’s just so convenient.

[Participant #3]

#### Theme 6: Efficient Data Sharing

Some participants requested an automated data-sharing feature across health care institutions to improve accessibility and coordination of care.

“Is there a way for an app to automatically share the data with hospitals and clinics? It would be really helpful for people who go to public clinics or get their medications from pharmacies. This way, hospitals and doctors would know what’s going on with the person. It would make things easier and faster for everyone.”

[Participant #6]

#### Theme 7: Interactive Design

It was suggested that mHealth apps should include a web-based consultation feature with experts for convenient advice on non-urgent issues. Additionally, some patients with DM proposed having a forum-like function to connect with others, share experiences, and discuss common challenges in managing their condition.

“Getting to the hospital isn’t always convenient for us. Having the option for online consultations with doctors through the app would be beneficial.”

[Participant #2]

“I think it would be really helpful if there was a forum feature in our app. It could allow us to connect with each other. When you chat with a fellow diabetic patient, you can learn and support each other. When we encounter unclear situations, suggestions from other patients can help us relax.”

[Participant #7]

## Discussion

### Principal Findings

Descriptive qualitative research was conducted with patients with DM in Hong Kong to understand the value and usefulness of various mHealth app features that may improve their self-management. Participants identified key features such as accurate information resources, automatic tracking and monitoring of health metrics, reminders, and personalized customization options. They also emphasized the importance of intuitive usability, efficient data-sharing capabilities, and an interactive design.

Participants highlighted the significance of providing clinically validated, comprehensive DM-related content within mHealth apps. They envisioned the app as a centralized, authoritative resource covering key self-care aspects, such as disease knowledge, prevention of comorbidities, exercise recommendations, and medication management. Beyond informational resources, participants emphasized the need for decision support to guide users in making informed choices about diet, fitness, and other lifestyle factors. This aligns with the broader challenge faced by many patients with DM who often lack access to professional, disease-specific knowledge, as existing web-based resources tend to be fragmented and unreliable [23]. This research emphasizes drawing upon evidence-based guidelines, current literature, focus groups, and interviews to develop a robust, validated knowledge base [24]. The involvement of users, developers, and clinical experts is considered essential during the app development stage, as this multilateral, user-centric approach is crucial for empowering patients to optimize their self-care regimens [25].

Participants also expressed a desire for the app to automatically track and monitor health metrics, with the ability to recognize and convert images into corresponding data for storage. Regular tracking of health is often regarded as a burden by patients with chronic diseases [26]. Knowing one’s history of physical activity and other health-related data through a tracking function was seen as beneficial and motivating [12]. This aligns with prior research showing that over 70% of health apps are designed to support healthy eating, with features like carbohydrate counting and diet tracking [27]. Automatically recognizing images and converting them into data that can be uploaded eliminates the need for manual tracking, which is a significant deterrent to the uptake of DM apps [16].

Participants claimed that reminder functions helped them be more aware of and accountable for their DM self-management activities, consistent with findings from prior studies [28-30]. Reminders and alerts can improve safety by warning of hypoglycemia or hyperglycemia and enhance self-management outcomes by improving medication adherence, physical activity, and clinical appointments [9]. However, current reminders are often ignored due to poor design and frequent pop-ups [13]. Regular self-care behaviors are crucial for effective DM management, and a recent study found that nearly a quarter of individuals with DM report frequently forgetting to take their DM medication [31]. Including a customizable and attention-grabbing reminder function in a self-management app is therefore vital.

Consistent with prior research [10], this study found that formulating corresponding personalized management strategies based on the user’s health assessment data is significant for a self-management app. The app’s ability to dynamically update its content and functionality based on the user’s evolving needs is a critical differentiator. For example, Duan et al [10] interviewed pregnant women with at least one risk factor for gestational DM who hoped that solutions tailored to their specific characteristics could encourage them to maintain healthy living habits during app use. Similarly, a qualitative study on Saudi women found that obese individuals were restricted from performing enough physical exercise outdoors [32]. The app should recommend personalized exercise routines aligned with the user’s conditions and preferences. Personalized features in health apps can improve user compliance with lifestyle interventions, one of the most common strategies in mHealth apps [33]. The app should leverage artificial intelligence (AI) algorithms to provide personalized information and recommendations, tailoring content, reminders, and feedback based on user preferences, blood glucose control, and self-management knowledge. Participants expect personalized feedback based on their tracked data and the ability to customize reminder frequency and timing based on their needs. Excessive reminders were deemed annoying [12].

Most participants emphasized that good usability design is crucial for the adoption and continued usage of mHealth apps, especially for older adults with DM and patients with lower overall literacy, whose reduced physical or cognitive capabilities can be obstacles when effectively using apps [13,34]. This includes using plain, simple language with explanations, providing multi-language options, and presenting information in visual forms such as illustrations and videos. These design elements are key for attaining health literacy, as suggested by the German guideline for evidence-based health information [35]. Previous studies have pointed out that multimedia content can better adapt to the learning styles of different individuals, enable patients to take a more active role in learning, and encourage them to study more actively [36]. Research has found that information retained from watching videos is stronger than that acquired from browsing text or static images alone [37]. Therefore, when designing apps, developers should use visual methods to present a variety of health information and educational resources for patients with DM to meet their needs for information support.

This study also underscores the need for cultural adaptation when developing DM mHealth apps for local patient populations. Tailoring the app’s content and features to align with users’ regional or ethnic cultural, lifestyle, and dietary habits can enhance satisfaction and engagement [38,39]. For instance, the app could incorporate region-specific calorie calculation functions, dialect support in voice recognition, and integration of traditional Chinese medicine principles such as syndrome differentiation and seasonal wellness [40]. Future application development should consider co-design approaches involving target users, inclusion of multilingual interfaces, and alignment of content with culturally accepted dietary and lifestyle practices to improve engagement and usability.

This literature demonstrates that in-app peer support and professional health coaching are appealing features for patients, aligning with previous findings that interactive functions are the most popular in mobile health apps [41,42]. Some studies have used telephone or messaging groups to support patients with DM [43,44]. However, these approaches often face challenges in providing timely feedback between providers and patients, and the information shared can be easily forgotten. Therefore, designing an interactive DM management mHealth app is crucial.

Such interactive apps can formulate targeted behavior change plans for users, continuously monitor their behavioral patterns, and provide personalized feedback, effectively motivating users to complete desired self-management behaviors [45,42,46]. The literature indicates that 2-way mHealth communication between health care providers and patients may enhance glucose monitoring, patient adherence, and clinical outcomes, such as reduced HbA_1c_ levels [47]. Additionally, peer support is crucial for developing and sustaining patients’ self-management skills, as it allows for sharing experiences, attitudes, and coping strategies, increasing their ability to manage the stress of self-care [48,49]. Collectively, participants with DM recommended including both provider-patient communication and peer support in apps, which is worth considering by app developers [46].

To maintain innovation and competitiveness in the evolving mHealth landscape, apps informed by this study should incorporate adaptive features that reflect both user needs and technological advancements. Integrating AI-driven personalization, culturally tailored content, and co-designed interfaces with patients can differentiate such tools from generic solutions [50]. Moreover, building in mechanisms for real-time user feedback, continuous performance analytics, and agile updates will enable ongoing refinement in response to emerging trends. Collaborations with interdisciplinary teams—including health care professionals, technologists, and patient advocates—will be essential to ensure sustained relevance and market adaptability.

While AI-powered mHealth apps hold promise for delivering personalized diabetes care, their implementation presents several challenges that warrant consideration. Key concerns of the participants include algorithmic transparency, clinical validity, and data privacy [42]. To ensure safety and reliability, AI tools must undergo rigorous testing against established clinical benchmarks, with transparent reporting of algorithm design and performance metrics. Regular auditing and bias detection mechanisms should also be incorporated to maintain fairness and accuracy across diverse patient populations. In addition, privacy-preserving techniques—such as federated learning and differential privacy—may be adopted to protect patient data while enabling the development of adaptive, intelligent systems. These considerations are critical for future implementation and warrant further investigation through interdisciplinary research that integrates technical feasibility, ethical safeguards, and patient trust.

### Strengths and Limitations

Previous research has primarily concentrated on users’ experiences and evaluations of developed apps after they have been used, with limited user involvement in the design process. This gap can lead to suboptimal user retention. In this study, we conducted in-depth interviews to investigate the preferences and functional needs of mHealth apps among individuals with DM. The insights gained are crucial for informing the development of future interventions and apps.

Additionally, we conducted thorough interviews with patients with DM in Hong Kong, where there is currently a lack of relevant research. Recognizing the importance of cultural adaptation for the success of mHealth apps, our findings can contribute to enhancing engagement among Hong Kong patients, thereby improving self-management levels and reducing health care costs. Furthermore, we emphasized the importance of using AI to provide personalized, timely, and culturally relevant information and feedback, which can also alleviate the workload of health care professionals. Ensuring the credibility and professionalism of AI information sources is essential to avoid misunderstandings that could delay treatment.

This study has several limitations. First, this study is limited by its relatively small sample size and recruitment from a single community health center, which may affect the generalizability and diversity of findings. Patients with different ages, disease durations, severity levels, and lifestyles may have distinct needs for mHealth apps, which were not fully captured. In addition, perspectives from health care professionals, such as diabetologists and diabetes educators, were not included, representing an important avenue for future research. Future studies should aim to recruit larger and more diverse samples from both community and hospital settings. Second, we were unable to capture data on how self-management experiences and perceptions of mHealth might differ across various identities (such as gender, age, and ethnicity), which could have provided richer insights into these subgroups. Third, while patients’ preferences for mHealth app features were explored qualitatively, the study did not adopt formal prioritization methods. Future studies should consider using structured prioritization techniques to systematically capture and rank patient preferences across diverse populations.

This study did not evaluate the actual behavioral changes or clinical health outcomes following mHealth app use, which limits our ability to assess the real-world impact of the proposed features. As the design phase preceded implementation, outcomes such as glycemic control, treatment adherence, and lifestyle modifications were not measured. This limits the ability to draw conclusions about the long-term effectiveness of such apps in supporting self-management.

### Conclusions

Our findings highlight the importance of tailoring these apps to local cultures, particularly through the integration of local food options, recommendations, and language preferences. Key features desired to enhance self-care behaviors include credible resources, automatic tracking and monitoring of health metrics, reminders, personalized customization, user-friendliness, efficient data-sharing capabilities, and in-app support via virtual interactions with peers and health care professionals. The results of this study provide valuable insights for enhancing the self-management capabilities of patients with DM and for the future development of mHealth apps aimed at DM prevention.

## Abbreviations

DM: diabetes mellitus
mHealth: mobile health
